# Causal associations of thyroid function and sudden sensorineural hearing loss: a bidirectional and multivariable Mendelian randomization study

**DOI:** 10.3389/fneur.2023.1269545

**Published:** 2023-11-27

**Authors:** Jialei Chen, Chao Wu, Jing He, Linsui Wu, Yongkang Yang, Shixun Zhong, Jing Luo

**Affiliations:** ^1^Department of Otolaryngology, The First Affiliated Hospital of Chongqing Medical University, Chongqing, China; ^2^Department of Pathology and Pathophysiology, Chongqing Medical University, Chongqing, China; ^3^Molecular Medicine and Cancer Research Center, College of Basic Medical Sciences, Chongqing Medical University, Chongqing, China; ^4^Department of Radiology, The First Affiliated Hospital of Chongqing Medical University, Chongqing, China; ^5^Department of Clinical Medicine, Clinical Medical College of Chengdu University, Chengdu, China; ^6^Department of Neurology, The First Affiliated Hospital of Chongqing Medical University, Chongqing, China; ^7^Department of Pathology, Chongqing Medical University, Chongqing, China

**Keywords:** sudden sensorineural hearing loss, free thyroxine, thyroid-stimulating 2 hormone, risk factor, Mendelian randomization

## Abstract

**Background:**

Observational studies have indicated a potential association between thyroid dysfunction and the risk of sudden sensorineural hearing loss (SSNHL). However, the precise causal relationship between the two remains uncertain. The objective of our study was to assess the causal influence of thyroid function on SSNHL by employing a bidirectional and multivariable Mendelian randomization (MR) approach.

**Methods:**

Single-nucleotide polymorphisms (SNPs) associated with free thyroid (FT4) and thyroid stimulating hormone (TSH) were selected from the summary data of a large genome-wide association study (GWAS) conducted on European individuals. The summary-level data of SSNHL were also obtained from a GWAS, which included 196,592 participants (1,491 cases and 195,101 controls). The MR analysis primarily utilized the inverse variance weighted (IVW) method, with sensitivity analyses performed using the weighted median, MR-Egger, and MR-PRESSO approaches.

**Results:**

In the IVW method, an elevated genetically predicted FT4 level was found to effectively reduce the risk of SSNHL (OR = 0.747, 95% CI = 0.565–0.987, *P* = 0.04). These findings were consistent when conducting multivariate MR analysis, which adjusted for TSH levels (OR = 0.929, 95% CI = 0.867–0.995, *P* = 0.036). However, genetically predicted TSH levels did not emerge as a risk factor for SSNHL (OR = 1.409, 95% CI = 0.895–1.230, *P* = 0.547). Furthermore, even after adjusting for FT4 levels in the multivariate MR analysis, no evidence of a direct causal relationship between TSH levels and the risk of SSNHL was observed (OR = 1.011, 95% CI = 0.880–1.161, *P* = 0.867). The reverse MR analysis showed that there was no evidence of a direct causal relationship between SSNHL and the risk of FT4 level (OR = 1.026, 95% CI = 0.999–1.054, *P* = 0.056) or TSH level (OR = 1.002, 95% CI = 0.989–1.015, *P* = 0.702).

**Conclusion:**

Within the normal range, genetic variants associated with higher FT4 levels demonstrate a potential protective effect against SSNHL, whereas there is no direct causal relationship between TSH levels and the risk of SSNHL.

## 1 Introduction

Sudden sensorineural hearing loss (SSNHL) is a common and alarming otolaryngological emergency with unknown etiology. It was defined as a sudden occurrence of unexplained sensorineural hearing loss occurring within 72 h, including hearing loss greater than 30 dB affecting at least in three consecutive frequencies (1). The incidence of SSNHL in Western countries ranges from 5 of 100,000 to 400 of 100,000 (2–4). The latest research reported that autoimmune diseases (5), infectious diseases (6), vascular diseases (7), and viral infections (8) are the most common causes of SSNHL, which indicates that SSNHL is caused by many factors (systemic and local).

The incidence rate of SSNHL in the worldwide is gradually rising. The World Health Assembly estimated that more than 2.5 billion people worldwide will be living with hearing loss to varying degrees by 2050 (9). Hearing loss not only leads to numerous neurological and psychological ailments but also significantly diminishes the quality of life for affected individuals, resulting in reduced productivity and an escalating social burden (10). Therefore, the swift and effective establishment of practical prevention and treatment strategies holds the utmost importance for otologists.

Thyroid hormone plays a vital role in the developmental maturation of hair cells spiral and ganglion cells as well as in the metabolism of the vascular cortex and stria vascularis (11–14). Thyroid dysfunction (hypothyroidism and hyperthyroidism) has been associated with increased hearing thresholds, abnormal V wave, and TOAE in auditory brainstem responses (15, 16). Additionally, there is evidence that SSNHL patients suffered from a higher prevalence of thyroid disease in comparison to the general population (17). However, it is important to note that these studies are based on clinical observations, which may introduce potential selection biases, confounding factors, and the possibility of reverse causality. Consequently, the causal relationship between thyroid function and SSNHL remains an unresolved question. By elucidating the causal connection between thyroid function and SSNHL, effective prevention and treatment strategies can be developed for the benefit of SSNHL.

The Mendelian randomization (MR) investigates causal relationships between risk factors associated with diseases using genetic variants as instrumental variables (IVs) (18). This emerging epidemiological technique effectively mitigates potential confounding factors and interferences, enabling more robust causal conclusions compared to traditional observational studies (19). Previous research utilizing MR analysis has successfully demonstrated causal relationships between FT4 and TSH levels with C-reactive protein (20), age-related macular degeneration (AMD) (21), and atrial fibrillation (22). Building upon this foundation, our study utilizes a large-scale genome-wide association study (GWAS) to examine the causal relationship and risk between thyroid function and SSNHL by using a bidirectional and multivariable MR analysis. By doing so, we aspire to contribute fresh perspectives and insights into the etiology of SSNHL.

## 2 Method

### 2.1 Study design

**Figure 1:**
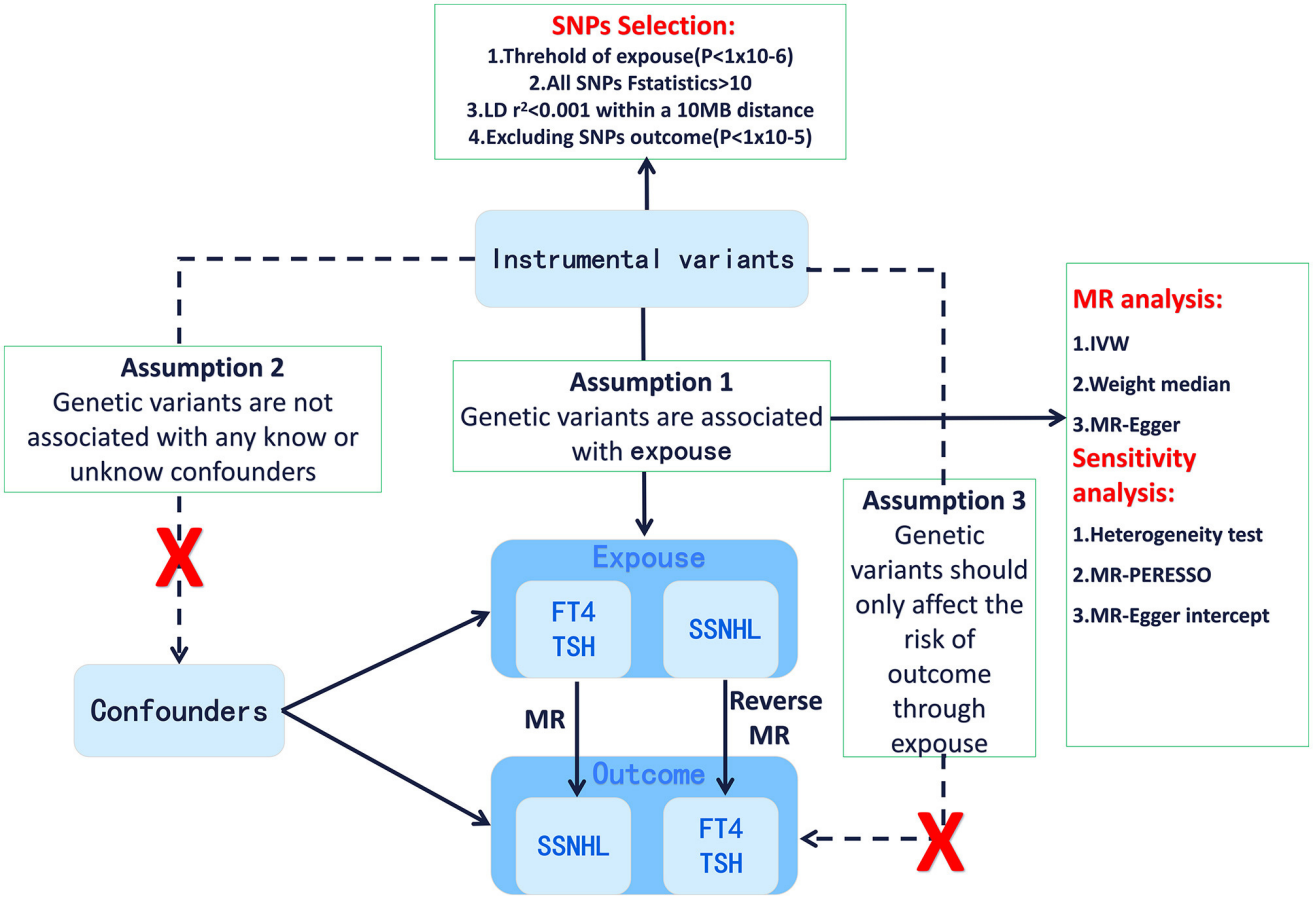
Framework design for the thyroid function-SSNHL two-sample bidirectional MR analyses. SSNHL, sudden sensorineural hearing loss; MR, Mendelian randomization; FT4, free thyroxine; TSH, thyroid-stimulating hormone.

Utilizing a bidirectional and multivariable Mendelian randomization analysis, we aimed to explore the potential causal relationship between genetically predicted TSH levels and FT4 levels and their association with SSNHL. A robust MR design relies on three fundamental assumptions: (1) The correlation hypothesis, which assumes a strong correlation between genetic variation and the exposure factors (thyroid function). (2) The independence hypothesis, which posits that genetic variation is independent of confounding factors that may influence both the exposure and outcome. (3) The exclusivity hypothesis, which suggests that genetic variation only impacts the outcome (SSNHL) through exposure and not through alternative pathways (23). Figure 1 provides an overview of the design employed in this thyroid function-SSNHL two-sample bidirectional MR study. As this study involves a reanalysis of previously published data, no additional ethical approval is required.

### 2.2 GWAS data of thyroid function

The genetic association of TSH within the reference range was obtained from the GWAS meta-analysis conducted by Zhou et al., encompassing a total of 119,715 subjects from the Nord-Trøndelag Health Study, Michigan Genomics Initiative, and the ThyroidOmics consortium, with over 22.4 million genetic markers. These data are available for download from the GWAS database (https://www.ebi.ac.uk/gwas) (24). The summary data of FT4 within the reference range was derived from the GWAS meta-analysis carried out by Teumer et al. that involved 49,269 individuals and more than 8 million genetic markers. These data can be accessed for download from the dbGaP website with the accession number phs000930 (25).

### 2.3 GWAS data of SSNHL

Genetic data pertaining to SSNHL was obtained from the publicly accessible GWAS database, specifically identified with the entry number “finn-b-H8_HL_IDIOP.” The study encompassed a total of 196,592 participants, consisting of 1,491 cases and 195,101 controls.

### 2.4 Instrumental variable selection

Based on the GWAS results for TSH and FT4, we conducted a rigorous screening of single nucleotide polymorphisms (SNPs) that exhibited close associations with TSH and FT4, achieving genome-wide significance (*P* < 5 × 10^−8^). SNPs closely associated with SSNHL were defined by the criterion of a *P*-value of <10^−6^. These selected SNPs were then utilized as instrumental variables (IVs) in the Mendelian randomization (MR) analysis. The IVs for MR analysis were chosen based on the following criteria: (1) To mitigate estimation bias resulting from weak IVs, we employed the equation *F* = (*R*^2^ × (*n* – 2))/(1 – *R*^2^) to assess the correlation between instrument strength and exposure. A significant correlation was considered when *F* ≥ 10. The estimated *R*^2^ of IVs was calculated using the equation 2EAF (1 – EAF)^*^β^2^, where EAF denotes the frequency of the effect allele and β represents the estimated genetic impact on FT4 (or TSH) (26). (2) To account for the influence of linkage disequilibrium, we ensured that the *r*^2^-value was less than 0.001 within a distance of 10MB (27). (3) In order to satisfy the exclusive hypothesis (that IV variants solely affect SSNHL through thyroid function), any hearing loss-related SNP outcomes (*P* < 1 × 10^−5^) were excluded from each analysis as well. Phenoscanner search was used to eliminate all known phenotypes associated with any genetic instruments considered in our analysis (28). In Supplementary Table 1, we summarized the association between exposure and SNPs and their relationship to outcome.

### 2.5 Univariate Mendelian randomization analysis

The primary analysis employed to assess the causal relationship between the exposure and outcomes is the inverse variance weighting (IVW) method. This method involves regressing the genetic variance (exposure) of TSH and FT4 against the genetic variance (outcome) of SSNHL, with each data point representing a conflict (29). However, it is important to note that the estimated effect obtained through IVW may be subject to bias. To address this, we conducted additional sensitivity analyses using MR-Egger and weighted medians as supplementary approaches to IVW (30). The intercept derived from the MR-Egger regression model serves as an indicator of directional pleiotropy, whereby a *p*-value below 0.05 suggests the presence of horizontal pleiotropy (31). Weighted median estimates generally provide robust estimates that are nearly as accurate as those obtained through IVW even in situations where more than half of the genetic variants violate assumptions (32). The MR-PRESSO method detects and eliminates outliers to yield relatively unbiased estimates while also identifying potential horizontal pleiotropic effects of SNPs through global testing (33). We utilized Cochran's *Q*-test to assess the heterogeneity of all SNPs. Additionally, employing the leave-one-out method, we systematically removed each SNP one at a time to evaluate whether bias in MR estimation is driven by a single SNP by calculating the causal effect of gene-predicted exposure on the outcomes using the remaining SNPs (33).The reverse MR analysis was conducted with the objective of exploring whether SSNHL might be a risk factor for FT4 levels or TSH levels. Given that genotypes are established at conception in accordance with Mendelian segregation laws, the potential for reverse causality is greatly reduced (34).

### 2.6 Multivariable Mendelian randomization analysis

The function of multivariate MR is similar to the independent evaluation of the effect of several intervention modalities in a randomized controlled trial. For this approach, genetic tools may be associated with multiple risk factors, but they must meet the equivalent instrumental variable assumption (35, 36). To investigate the independent influence of FT4 and TSH on the risk of SSNHL, given their close correlation, we employed multivariate MR analysis. When MR analysis showed a causal relationship between FT4 (TSH) and SSNHL, multivariate MR analysis was performed to evaluate the role of TSH (FT4) as a risk factor for SSNHL. The SNPs used in the multivariate MR analysis were derived from the combination of instrumental variables (IVs) identified in the univariate MR analysis for each exposure (35). To ensure data quality, we limited our analysis to SNPs with a clumping threshold of *r*^2^ <0.001 within a 10 MB region and removed any duplicates. A *p*-value less than 0.05 was considered statistically significant when estimating the causal effect of exposure. All statistical analyses were performed using the R package “TwoSampleMR2 (version 0.5.6)” in R (version 4.2.1). For further details, please refer to the following link: https://mrcieu.github.io/TwoSampleMR/ (37).

## 3 Result

### 3.1 Causal association of FT4 with SSHNL

**Figure 2:**
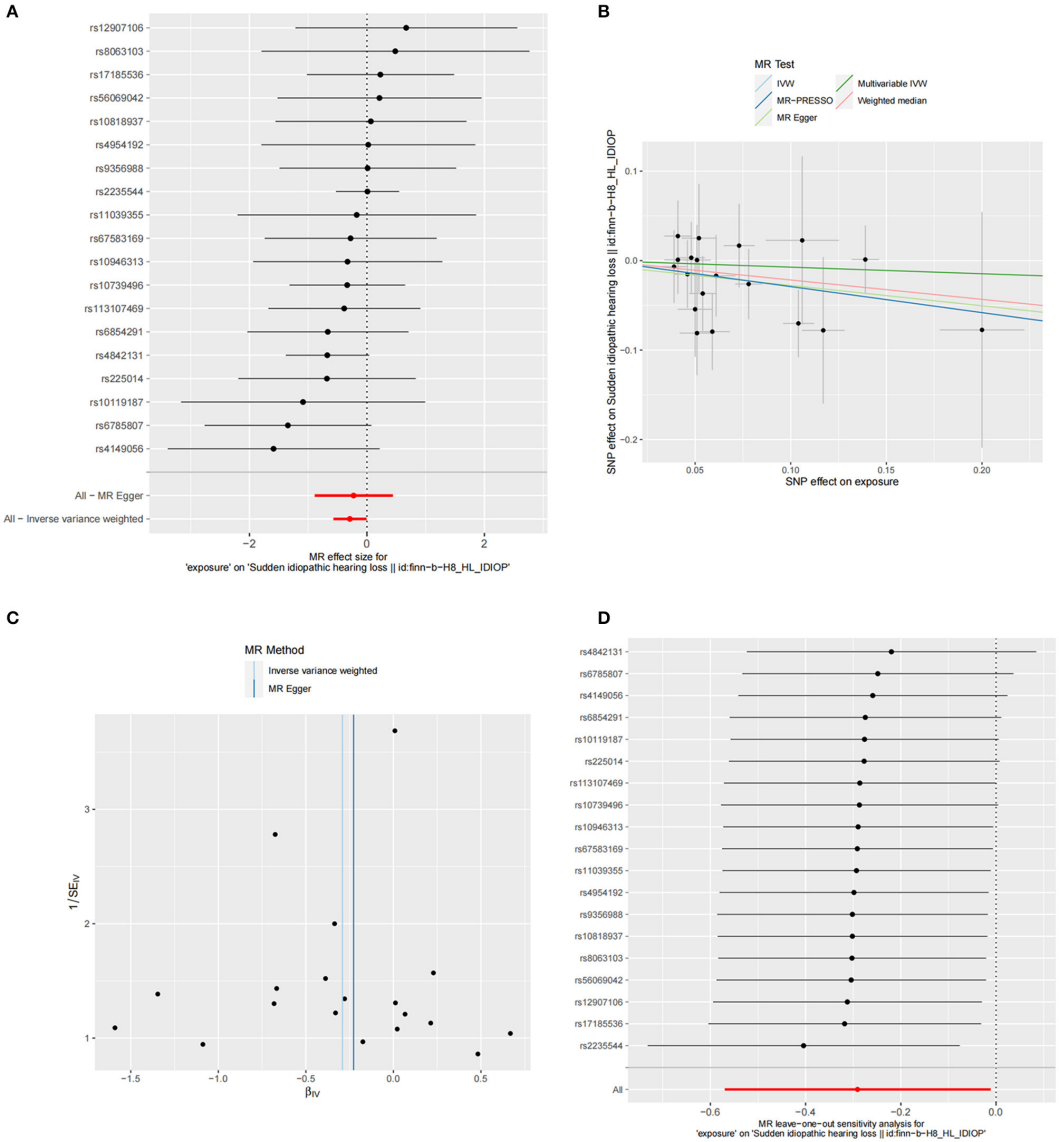
**(A)** Forest plot of the potential effects of FT4-associated SNPs on SSNHL. **(B)** Scatter plot demonstrates the effect of each FT4-associated genetic variant on SSNHL on the log-odds scale. **(C)** Funnel plot of the causal effect of FT4-related SNPs on SSNHL. **(D)** Leave-one-out plots for the MR analyses of SSNHL on FT4.

The summary statistics of SSNHL include all 19 SNPs associated with FT4 levels. The F-statistic for each of the SNPs included in the analysis exceeded 10 (FT4 F statistics ranged from 32.66 to 479.82). Phenoscanner analysis showed that there was no association between SNPs and any other traits that could confound the exposure-outcome relationship. The MR analysis using the IVW method revealed a significant causal relationship between FT4 levels and the risk of SSNHL (OR = 0.747, 95% CI = 0.565–0.987, *P* = 0.04). The forest plot illustrates that genetically predicted FT4 levels are significantly associated with SSNHL (Figure 2A). Similarly, risk estimation results in MR-Egger regression and weighted median methods demonstrate a similar trend although the associations did not reach statistical significance (Figure 2B; Table 1). The *P*-values obtained from the Cochran *Q*-test for MR-Egger (Cochrane's *Q* = 10.54, *P* = 0.88) and IVW (Cochrane's *Q* = 10.50, *P* = 0.91) were both greater than 0.05, indicating no heterogeneity in the results (Figure 2C). No abnormal instrumental variables were found to contribute to pleiotropic effects in the overall MR estimation, as indicated by the global test for MR-PRESSO (*P* Global Test = 0.88). The leave-one-out sensitivity analysis suggests that the overall impact of FT4 on SSNHL is not driven by a single SNP (Figure 2D). We conducted a reverse MR analysis to assess the causal impact of SSNHL on FT4 levels. Following the application of the aforementioned criteria, we identified 14 SNPs significantly associated with SSNHL (Supplementary Table 1). In our reverse MR analysis employing the IVW method, we found no significant evidence supporting a causal relationship between SSNHL and the risk of FT4 levels (OR = 1.026, 95% CI = 0.999–1.054, *P* = 0.056; Supplementary Table 2). Finally, the results of the multivariate MR analysis, with adjustments made for TSH levels using the IVW method, demonstrated a direct causal effect of FT4 levels on the risk of SSNHL (OR = 0.929, 95% CI = 0.867–0.995, *P* = 0.036).

**Table 1:** MR results of FT4 and TSH on risk of SSNHL.

**Exposure**	**Method**	**No. of SNPs**	**OR**	**(95% CI)**	***P*-value**
FT4	IVW	19	0.747	(0.565–0.987)	0.040
	Weighted median	19	0.805	(0.553–1.171)	0.257
	MR-Egger	19	0.796	(0.411–1.542)	0.509
	MR-PRESSO	19	/	/	0.886
	Multivariable IVW	19	0.929	(0.867–0.995)	0.036
TSH	IVW	88	1.409	(0.895–1.230)	0.547
	Weighted median	88	1.108	(0.860–1.427)	0.402
	MR-Egger	88	0.900	(0.647–1.252)	0.536
	MR-PRESSO	88	/	/	0.905
	Multivariable IVW	87	1.011	(0.880–1.161)	0.867

SSNHL, sudden sensorineural hearing loss; MR, Mendelian randomization; FT4, free thyroxine; TSH, thyroid-stimulating hormone; IVW, inverse variance weighted; SNPs, single-nucleotide polymorphisms; MR-PRESSO, Mendelian randomization-pleiotropy residual sum outlier; OR, odds ratio; multivariable MR using IVW of FT4 and TSH on SSNHL risks.

### 3.2 Causal association of TSH with SSHNL

**Figure 3:**
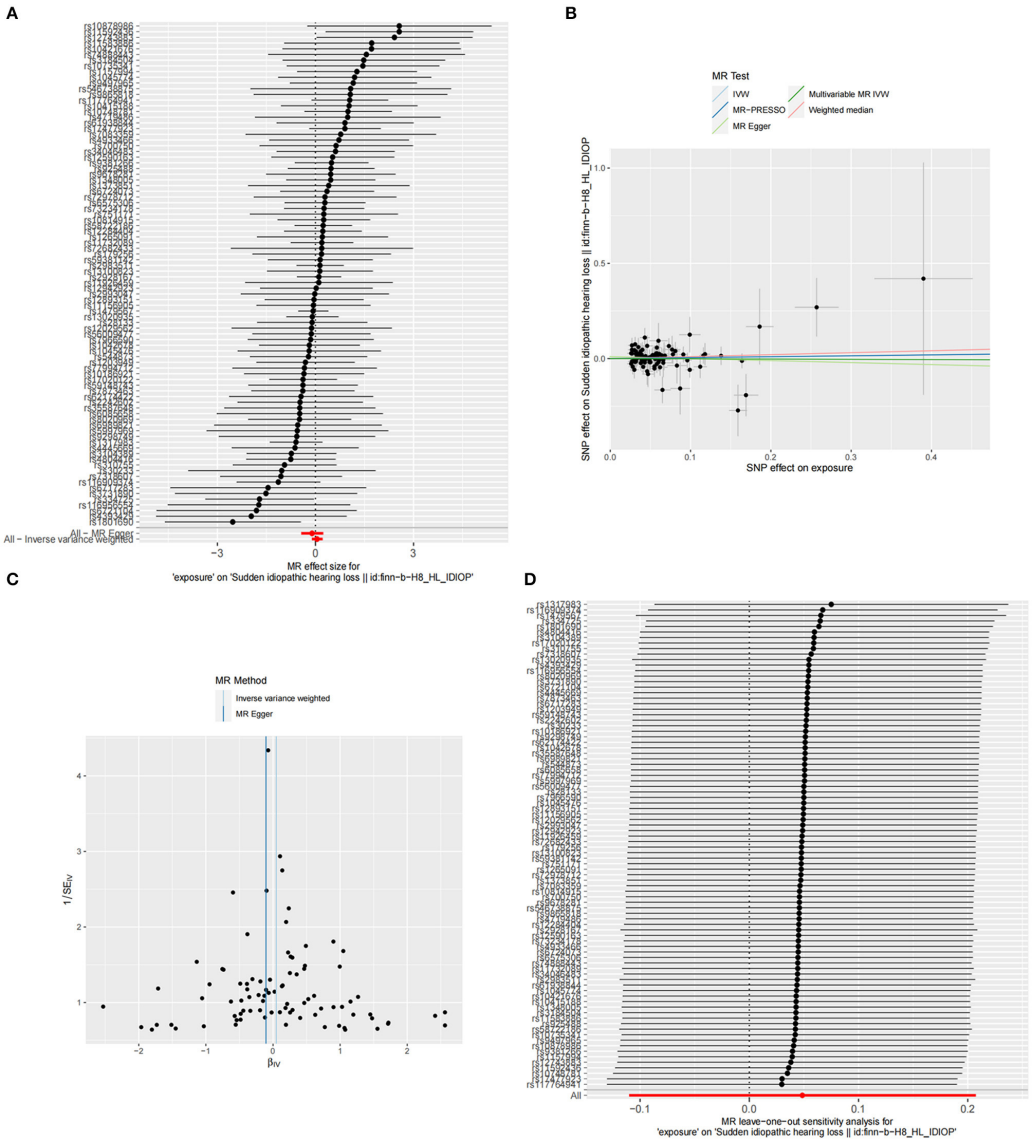
**(A)** Forest plot of the potential effects of TSH-associated SNPs on SSNHL. **(B)** Scatter plot demonstrates the effect of each TSH-associated genetic variant on SSNHL on the log-odds scale. **(C)** Funnel plot of the causal effect of TSH-related SNPs on SSNHL. **(D)** Leave-one-out plots for the MR analyses of SSNHL on TSH.

The summary statistics of SSNHL include all 88 SNPs associated with TSH levels, each with an F-statistic greater than 10 (TSH F statistics ranged from 37.00 to 1541.78). However, we did not find significant evidence indicating a potential causal effect of TSH on the risk of SSNHL (IVW, OR = 1.409, 95% CI = 0.895–1.230, *P* = 0.547; Figure 3A). Similar risk estimation results were observed in the MR-Egger regression and weighted median methods. The Cochran *Q*-test results for MR-Egger (Cochrane's *Q* = 69.39, *P* = 0.90) and IVW (Cochrane's *Q* = 70.47, *P* = 0.90) yielded *p*-values greater than 0.05, indicating no heterogeneity in the results (Figure 3B; Table 1). No abnormal instrumental variables were identified that could lead to pleiotropic effects in the overall MR estimation, as indicated by the global test for MR-PRESSO (*P* Global Test = 0.88; Figure 3C). In the leave-one-out sensitivity analysis, it was determined that the association between TSH and SSNHL was not driven by a single SNP (Figure 3D). We conducted a reverse MR analysis using the IVW method, and the results did not yield significant evidence indicating a potential causal effect of SSNHL on TSH levels (OR = 1.002, 95% CI = 0.989–1.015, *P* = 0.702; Supplementary Table 2). Furthermore, even in the multivariate MR analysis, where adjustments were made for FT4 levels, we found no evidence supporting a direct causal relationship between TSH levels and the risk of SSNHL (IVW, OR = 1.011, 95% CI = 0.880–1.161, *P* = 0.867).

## 4 Discussion

Based on the comprehensive large-scale GWAS summary statistics, our bidirectional and multivariable MR study revealed a negative correlation between FT4 levels and the risk of SSNHL. However, there is insufficient evidence to support a significant association between TSH levels and the risk of SSNHL. To investigate the independent influence of FT4, we conducted a multivariate MR analysis to account for any potential interaction between FT4 and TSH, and the results remained consistent.

Thyroid hormone is an essential endocrine substance that plays a critical role in the development of the auditory system (38). The middle and inner ears are highly sensitive to fluctuations in thyroid hormone serum levels (39). Thyroid hormone plays a vital role in the development and maturation of spiral ganglion cells and hair cells as well as in the metabolism of the vascular cortex and stria vascularis (11–14). Both hypothyroidism and hyperthyroidism have the potential to cause sensorineural hearing loss, that may be manifested as intracochlear, posterior cochlear, or central hearing impairments (40). Moreover, the blood supply to the cochlea primarily depends on a single labyrinthine artery with no collateral circulation. Individuals with hypothyroidism may experience hypercoagulability, thereby increasing the risk of thromboembolism (41). Hair cells, which consume a significant amount of oxygen, are highly susceptible to hypoxia, which can result in hair cell damage. While reports on the correlation between TSH and SSNHL are limited, a recent study underscores the significance of early TSH testing in the diagnosis of SSNHL (42). This study identified early lower or abnormal TSH levels as independent predictive factors for moderate-to-severe SSNHL, while FT4 level abnormalities were not a risk factor for SSNHL. Conversely, another study conducted a retrospective analysis of 676 SSNHL patients, and the results indicated that FT4 level disturbances were a risk factor for SSNHL, while TSH level abnormalities were not associated with an increased risk of SSNHL (43). This discovery is consistent with our findings. Potential reasons for this discrepancy may include those as follows: (1) gender-specificity in the correlation between SSNHL (44) and thyroid hormones as well as TSH levels (45). (2) SSNHL may be categorized into a minimum of four distinct subtypes, each characterized by unique pathogenic mechanisms (4). To the best of our knowledge, there is currently no study that has employed both gender-specific and subgroup-specific data to discuss the correlation between thyroid hormones, TSH, and the risk of SSNHL occurrence. Although a meta-analysis of the association between FT4 and SSNHL is currently lacking, several independent studies have suggested an association between hypothyroidism and susceptibility to SSNHL. These studies have demonstrated that hypothyroidism and hyperthyroidism are associated with SSNHL susceptibility (39). Additionally, research has shown positive correlations between hypothyroidism and risk of SSNHL in both young and elderly subgroups of patients (46). Furthermore, retrospective analysis of a large cohort of SSNHL patients revealed abnormal thyroid function test results in a substantial proportion of cases (43). Notably, the incidence of thyroid dysfunction in SSNHL patients was found to be more than twice that of the general population (17). Overall, these studies collectively support the pivotal role of FT4 in the occurrence and development of SSNHL.

The research design offers distinct advantages. First, it leverages freely accessible GWAS data, thereby significantly reducing research costs. Second, employing genetic variation as instrumental variables in MR analysis effectively mitigates confounding biases and reverse causal effects. Finally, our findings indicate that genetically predicted elevated levels of FT4 are associated with a reduced risk of SSNHL, offering potential clinical prevention strategies for otologists. Nonetheless, it is essential to acknowledge several potential limitations in our study. First, the study population primarily comprises individuals of European ancestry, prompting cautious interpretation of the generalizability of our findings to other populations. Second, thyroid function exhibits gender specificity, and unfortunately, due to limitations in available TSH and FT4 summary data, we were unable to conduct sex-specific MR analysis. Finally, there are at least four subgroups of SSNHL with different pathogenic mechanisms. However, we were also unable to perform subgroup-specific MR analyses due to the limitations of the available SSNHL summary data. Future research endeavors encompassing diverse populations and accounting for gender-specific and subgroup-specific effects would enhance our understanding of the causal relationship between thyroid function and SSNHL.

In summary, our bidirectional and multivariable MR analysis revealed that higher FT4 levels are associated with a decreased risk of SSNHL. However, we did not find evidence of an independent causal relationship between TSH and SSNHL risk. These findings contribute to our understanding of the relationship between thyroid function and SSNHL, offering new insights. We anticipate that our results will inform otologists of clinical prevention and treatment strategies for SSNHL.

## Data availability statement

The original contributions presented in the study are included in the article/Supplementary material, further inquiries can be directed to the corresponding authors.

## Ethics statement

Ethical approval was not required for the study involving humans in accordance with the local legislation and institutional requirements. Written informed consent to participate in this study was not required from the participants or the participants' legal guardians/next of kin in accordance with the national legislation and the institutional requirements.

## Author contributions

JC: Conceptualization, Resources, Supervision, Validation, Visualization, Writing—original draft, Writing—review & editing, Data curation, Investigation, Methodology. CW: Data curation, Resources, Validation, Visualization, Writing—original draft. JH: Resources, Validation, Visualization, Writing—original draft. LW: Resources, Validation, Visualization, Writing—original draft. YY: Resources, Visualization, Writing—original draft, Data curation. SZ: Conceptualization, Project administration, Writing—review & editing, Funding acquisition. JL: Conceptualization, Project administration, Writing—review & editing, Resources, Supervision, Validation, Visualization, Writing—original draft.
