# Supplementary material for: Causal associations of thyroid function and sudden sensorineural hearing loss: a bidirectional and multivariable Mendelian randomization study
Source: Front Neurol. 2023 Nov 27;14:1269545. doi: 10.3389/fneur.2023.1269545 (PMC10715417; doi:10.3389/fneur.2023.1269545)
Supplement: Supplementary file 2 [file Table_1.DOCX]

**Table S2**. The Reverse MR Results of SSNHL on Risk of FT4 and TSH

| **Exposure** | **Outcome** | **Method** | **No. of SNPs** | **OR** | **(95%CI)** | **P-value** |
| --- | --- | --- | --- | --- | --- | --- |
| SSNHL | FT4 | IVW | 9 | 1.026 | (0.999-1.054) | 0.056 |
|  |  | Weighted median | 9 | 1.026 | (0.553-1.171) | 0.065 |
|  |  | MR-Egger | 9 | 1.017 | (0.974-1.062) | 0.457 |
|  |  | MR-PRESSO | 9 | / | / | 0.34 |
| SSNHL | TSH | IVW | 13 | 1.002 | (0.989-1.015) | 0.702 |
|  |  | Weighted median | 13 | 0.999 | (0.981-1.017) | 0.947 |
|  |  | MR-Egger | 13 | 0.994 | (0.975-1.017) | 0.576 |
|  |  | MR-PRESSO | 13 | / | / | 0.342 |

SSNHL, sudden sensorineural hearing Loss; MR,Mendelian randomization;

FT4, free thyroxine; TSH,thyroid-stimulating hormone; IVW,inverse variance weighted; SNPs,single-nucleotide polymorphisms; MR-PRESSO,Mendelian randomization−pleiotropy residual sum outlier; OR,odds ratio;
